# Influenza virus circulation in Cambodia

**Published:** 2011-01-10

**Authors:** Sek Mardy, Sovann Ly, Seng Heng, Monica Naughtin, Sirenda Vong, Paul Kitsutani, Chea Huch, Sareth Rith, Borann Sar, Chea Nora, Buth Sokhal, Chadwick Yasuda, Tom Wierzba, Nima Asgari, Sok Touch, Philippe Buchy

**Affiliations:** 1 Institut Pasteur in Cambodia, Phnom Penh, Cambodia; 2 Communicable Disease Control Department, Ministry of Health, Phnom Penh, Cambodia; 3 Influenza Division, National Center for Immunization and Respiratory Disease, Centers for Disease Control and Prevention, Atlanta, GA, USA; 4 Centers for Disease Control and Prevention, Cambodia Office, Cambodia; 5 World Health Organization, Phnom Penh, Cambodia; 6 National Institute of Public Health, Phnom Penh, Cambodia; 7 Naval Medical Research Unit 2, Phnom Penh, Cambodia

## Background

The Cambodian National Influenza Center (NIC) was established in August 2006 for the purpose of documenting the dynamics of influenza disease and to virologically characterize the circulating strains. To continuously monitor influenza activity, a hospital-based sentinel surveillance system for ILI (influenza-like illness) with a weekly reporting and sampling scheme was initially established in five sites in 2006. In addition, hospital-based surveillance of acute lower respiratory infection (ALRI) cases was established in 2 sites.

## Methods

The sentinel sites collected weekly epidemiological data from patients who fulfilled the ILI case definition, and took naso-pharyngeal specimens from 5 to 10 cases per week. Over 4100 respiratory samples were collected from hospitalized ALRI patients between 2007 and 2010. All samples were tested in the Virology Unit at the Institut Pasteur in Phnom Penh. Viral RNA was extracted and amplified by a multiplex RT-PCR detecting influenza A and influenza B virus simultaneously. Influenza A viruses were then subtyped and analyzed by hemagglutination inhibition assay. The susceptibility to neuraminidase inhibitor drugs (oseltamivir, and zanamivir) was determined using the NA-STAR kit (Applied Biosystems®). Genetic analyses targeting HA, NA, and M genes were conducted on strains selected randomly.

## Results

We observed that 5.8% (30 of 516), 7.7% (96 of 1250), 15.3% (212 of 1382), 15.2% (909 of 6011) and 1.4% (25 of 1791) of overall clinical specimens were positive for influenza virus in the years 2006, 2007, 2008, 2009 and 2010 respectively. Until 2009, H3N2 virus was always the predominant influenza A sub-type detected: 100% in 2006, 65.9 % in 2007, 81.3% in 2008. No influenza B viruses were isolated in 2006 but accounted for 57.7% and 34% of all influenza strains in 2007 and 2008, respectively. Pandemic H1N1 (H1N1pdm) was first detected in Cambodia in August 2009 and subsequently replaced the seasonal H1N1 strain as the most frequently detected subtype. However, H3N2 continued to co-circulate in significant proportions through November 2009. Antigenic analyses show that seasonal H1N1 belonged to the groups A/New Caledonia/20/1999-like in 2007 and A/Brisbane/59/2007-like in 2008. H3N2 belonged to A/Wisconsin/67/2005-like in 2006 and 2007, and to A/Brisbane/10/2007-like in 2008 and 2009. The influenza B strains drifted from B/Malaysia/2506/2004-like in 2007 to B/Florida/4/2006-like in 2008 and then to B/Brisbane/60/2008-like in 2009. Sequences of the M gene obtained from representative 2007-2008 H1N1 and H3N2 strains contained the S31N mutation associated with adamantanes resistance. Except for a single H1N1 strain detected in 2007, no reduction in the susceptibility to neuraminidase inhibitors was observed among the influenza virus circulating from 2007 to 2010. Each year, the peak of influenza circulation was observed mainly from August to November. The proportion of specimens collected from patients with ILI which tested positive for Influenza virus varied between 0% (in May) and 51% (in October). In contrast, only 5 to 10% of the ALRI specimens were positive for influenza virus during peak of seasonal transmission. Since 2007, 4 new human cases of influenza A/H5N1 infection have been identified but by different surveillance systems (event-based surveillance, fever study)

## Conclusion

Peak seasonal influenza activity in Cambodia occurred during the rainy season from August to November. Although Cambodia is a tropical country geographically located in the northern hemisphere, influenza activity has a southern hemisphere transmission pattern. Therefore, while the northern hemisphere vaccine may provide partial protection in Cambodia, the southern hemisphere vaccine is recommended. The drug susceptibility profile of Cambodian influenza strains revealed that neuraminidase inhibitors would be the drug of choice for influenza treatment and chemoprophylaxis in Cambodia, as adamantanes are no longer expected to be effective.

